# Super-resolution imaging with a cucurbituril-encapsulated fluorophore†

**DOI:** 10.1039/d4cc05274a

**Published:** 2024-11-04

**Authors:** Liza Briant, Jimmy Maillard, Alexandre Fürstenberg

**Affiliations:** a Department of Physical Chemistry, University of Geneva 1211 Genève 4 Switzerland alexandre.fuerstenberg@unige.ch; b Department of Inorganic and Analytical Chemistry, University of Geneva 1211 Genève 4 Switzerland

## Abstract

Red-emitting oxazine fluorophores are shown to bind to cucurbit[7]uril (CB[7]) with high affinity. Their fluorescence quantum yield and lifetime are thereby enhanced owing to shielding of the dyes from water. Using CB[7] as an imaging additive leads to a larger number of photons detected per molecule in super-resolution experiments with the dye ATTO655.

## Abstract 2

Red-emitting oxazine fluorophores for super-resolution imaging become brighter upon binding to cucurbit[7]uril.

## Graphical abstract

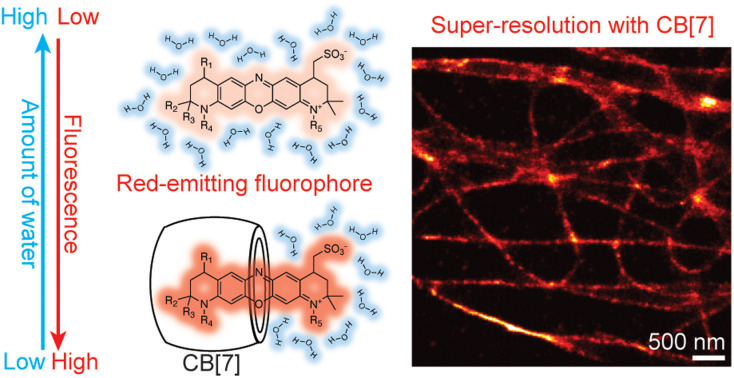

The development of single-molecule and super-resolution fluorescence microscopy has led to an active search for fluorescent dyes with perfected properties,^1^ and new schemes to improve the brightness and photostability of fluorophores are still in high demand.^2^ These parameters are critical in single-molecule and super-resolution fluorescence imaging as they define how many photons can ultimately be detected from a single emitter.^3^ The latter quantity directly relates to the localisation precision in single-molecule localisation microscopy (SMLM) schemes for super-resolution such as (d)STORM,^4,5^ PALM,^6,7^ or (DNA-)PAINT,^8,9^ and thereby to the achievable experimental resolution.^10^

In biological media, red excitation is usually preferred to minimise unwanted fluorescence from the sample or from potential impurities. However, red-emitting dyes often display low fluorescence quantum yields in aqueous environments^11–13^ due to specific fluorescence quenching by H_2_O in the contact sphere of the fluorophore.^13^ As biology mostly takes place in water, simple strategies to inhibit this particular quenching process would be beneficial to any form of fluorescence imaging.

We rationalised that molecular encapsulation of fluorophores should reduce their direct exposure to water and thereby increase their brightness. Formation of host–guest complexes between dyes and water-soluble macrocycles has led in several cases to significantly improved fluorescence properties.^14,15^ Cyclodextrins are popular macrocyclic hosts due to their high water solubility, but they bind dyes and other guests with rather low affinity (binding constant *K*_a_ ∼ 10^2^).^14^ We could recently observe a rise in fluorescence quantum yield and lifetime with some red-emitting fluorophores and attribute it to their shielding from water in their host–guest complex with cyclodextrins, but the association constant was too low to be of practical use.^16^

On the other hand, cucurbit[*n*]urils form another family of macrocyclic hosts made of *n* glycoluril units that display much larger affinities for their guests (*K*_a_ > 10^5^).^14^ Especially the more soluble (∼5 mM) cucurbit[7]uril (CB[7]) has been shown to efficiently bind a range of organic fluorophores, thereby improving their brightness, photostability, or solubility.^17–20^ We therefore set out to investigate the interaction between CB[7] and the red-emitting oxazines ATTO655, ATTO680, and ATTO700 (Fig. 1) that are used in SMLM^11,21–24^ and whose fluorescence properties are known to be sensitive to water.^11,13,16^

**Fig. 1:**
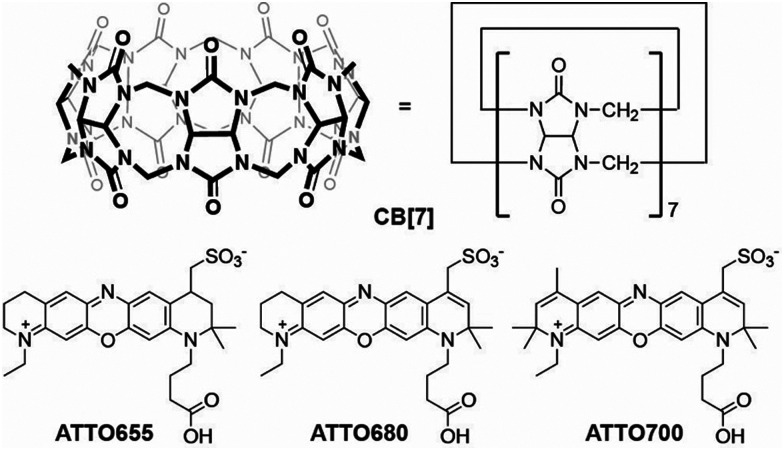
Molecular structure of CB[7] and of the dyes ATTO655, ATTO680, and ATTO700.

Addition of increasing concentrations of CB[7] to 1–2 μM solutions of each fluorophore in pure water led to spectral shifts in the absorption and emission spectra of the dyes indicative of their interaction with the macrocyclic host. Hypsochromic shifts of 5–8 nm in the absorption (Fig. 2a and Fig. S1a, ESI†) and bathochromic shifts of 4–8 nm in the emission (Fig. 2b and Fig. S1b, ESI†) occurred in the presence of a concentration of 1 mM of CB[7], while the molar absorption coefficient did not vary significantly, decreasing by *ca.* 5–15% with ATTO655 and ATTO680 (Fig. S2, ESI†). Most importantly, a CB[7]-dependent increase in the fluorescence quantum yield and in the excited-state lifetime (+34–48% at 1 mM CB[7]) was observed for all three fluorophores (Fig. 2b, c, e, Table 1 and Fig. S1, S3, ESI†), leading to an overall enhancement in their brightness in the complex.

**Fig. 2:**
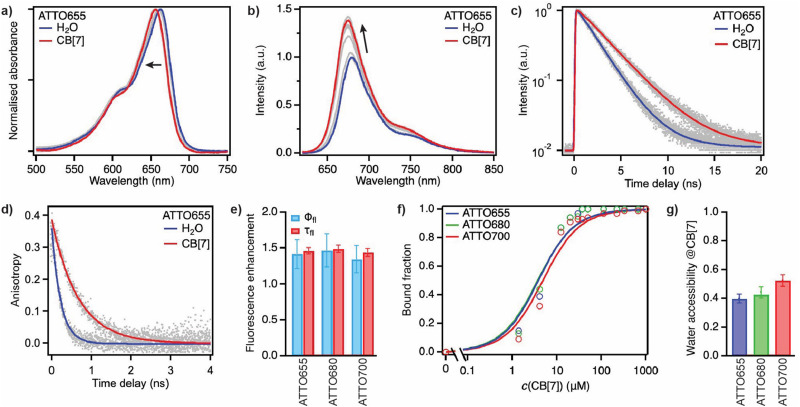
(a) Intensity-normalised absorption spectra and (b) fluorescence spectra of ATTO655 in pure H_2_O (blue traces) and in the presence of various concentrations of CB[7] (grey traces) up to 1 mM (red traces). (c) Fluorescence decays of ATTO655 in H_2_O and in the presence of 1 mM CB[7]. (d) Decay of the fluorescence polarisation anisotropy of ATTO655 in H_2_O and in the presence of 1 mM CB[7]. In (c) and (d), solid lines represent best monoexponential fits to the data points (grey). (e) Fluorescence quantum yield (blue) and fluorescence lifetime (red) enhancements (defined as *Φ*_fl_(CB[7])/*Φ*_fl_(H_2_O) and *τ*_fl_(CB[7])/*τ*_fl_(H_2_O), respectively) of the fluorophores in the presence of 1 mM CB[7] with respect to the free dyes in H_2_O. (f) Bound fraction of the investigated dyes as a function of the total CB[7] concentration. Solid lines represent fits to the data points (open circles) with a 1 : 1 binding isotherm. (g) Water accessibility of the investigated dyes bound to CB[7]. This parameter is equivalent to the residual quenching efficiency defined in ref. 16.

**Table 1:** Photophysical properties, dissociation constant, and relative hydration of the investigated fluorophores in pure H_2_O and in the presence of CB[7] (1 mM)

System		*Φ*_fl_ a	*τ*_fl_ b (ns)	*τ*_r_ c (ns)	*K*_d_/10^−6^	*f*_q_ d
ATTO655	H_2_O	0.28	1.91	0.28		1.00
	CB[7]	0.40	2.79	0.67	3.3 ± 0.6	0.40 ± 0.03
ATTO680	H_2_O	0.30	1.80	0.26		1.00
	CB[7]	0.44	2.68	0.67	3.4 ± 0.6	0.43 ± 0.04
ATTO700	H_2_O	0.25	1.64	0.30		1.00
	CB[7]	0.34	2.36	0.72	4.4 ± 0.8	0.52 ± 0.04

a Absolute fluorescence quantum yield measured using the free dyes in H_2_O as a standard.^13^

b Excited-state lifetime of the dye in free H_2_O (*τ*_1_ in Table S1, ESI) or when bound to CB[7] (*τ*_2_).

c Rotational correlation time extracted from the decay of the fluorescence polarisation anisotropy.

d Water accessibility of the dye in pure water (100%) and when bound to CB[7], estimated using eqn (S2) (see ESI).^16^

A host–guest interaction between the dyes and CB[7] was further supported by time-resolved fluorescence anisotropy measurements. The decay of the fluorescence polarisation anisotropy of the dyes was indeed significantly slower in the presence of 1 mM CB[7] compared to the free dyes in water and well reproduced by a single exponential component (Fig. 2d, Table 1 and Fig. S1c, ESI†). The measured rotational correlation times for these complexes are similar to those observed with the same dyes and cyclodextrins.^16^

The time decay of the fluorescence emission in the presence of various concentrations of CB[7] was analysed globally for each dye with a biexponential model accounting for two emissive, respectively free and CB[7]-bound dye populations (Fig. S3 and Table S1, ESI†). The lifetime of the free dye population was fixed in each case to the lifetime measured in H_2_O in the absence of host, whereas the lifetime of the bound-dye population was obtained from the analysis of samples at a 1 mM concentration of CB[7]. The amplitude of the bound-dye component and of the amplitude-weighted average lifetime increased, as expected, with increasing CB[7] concentration, reaching a plateau at ∼0.05 mM CB[7].

Fits to the lifetime binding data with a 1 : 1 binding isotherm yielded values for the thermodynamic dissociation constant *K*_d_ between 3 and 5 × 10^−6^ (Fig. 2f, Table 1 and Fig. S4, ESI†) for all dyes, numerically quite close to the used dye concentration (1–2 μM). Binding assays were thus performed almost in the stoichiometric regime,^25,26^ as also indicated by the experimental dose–response curves that are noticeably steeper than the fitted isotherms.^27^ These measurements thereby rather set a lower limit to the association constant *K*_a_ = 1/*K*_d_ of the dye-CB[7] complexes which must therefore be larger or equal to ∼2 × 10^5^, demonstrating a high affinity of the dyes for CB[7]. These values indicate significantly stronger binding of ATTO655, ATTO680 and ATTO700 to CB[7] than to cyclodextrins^16^ and are similar to those observed with other dye-CB[7] complexes.^14,19,28^ It is important to note that all assays were carried out in pure H_2_O as the solvent. Ions such as Na^+^ have indeed been shown to bind with high affinity to the portals of CB[7]^29^ and experiments performed in phosphate buffer saline (PBS) show reduced affinity binding of ATTO655 to CB[7] (*K*_a_ ∼ 8 × 10^3^) and limited CB[7] solubility (Fig. S5, ESI†).

Cucurbiturils are known to modulate the photophysics of encapsulated dyes by suppressing intra- or intermolecular non-radiative deactivation or changing the local polarity or polarisability.^18,30,31^ In the case of the investigated oxazine dyes, binding to CB[7] did not affect the radiative lifetime. The increase in fluorescence lifetime and fluorescence quantum yield can be interpreted as exclusion of water from the contact sphere of the dyes, which are otherwise rather insensitive to changes in their microenvironment.^13,16^ A comparison of the fluorescence lifetime of the dyes in the complex with the lifetime in pure H_2_O and in a completely quenching-free environment such as pure D_2_O enabled to extract the water accessibility *f*_q_ of the fluorophore (Table 1).^16,23^ We found that CB[7] is able to quite efficiently isolate the dyes from H_2_O, with an estimated 48–60% of the water contacts being removed (Fig. 2g). These values are significantly higher than with cyclodextrins or than when the fluorophores are attached to a protein,^16^ pointing to CB[7] as an efficient additive to prevent the quenching of the fluorescence of these dyes by water, especially for ATTO655 which is the most used among them in single-molecule applications.

In order to evaluate the benefits of the increased brightness on single-molecule imaging, we next covalently immobilised ATTO655 on a glass surface and measured the number of photons collected from individual emitters imaged in pure H_2_O or with 1 mM CB[7] (Fig. 3a), a concentration at which saturation binding is reached. As expected, the distribution of photons detected per localised emitter and per frame was broader in the presence of CB[7] than in pure H_2_O and was shifted to higher values, with 2200 ± 910 photons detected with CB[7] and 1750 ± 590 in H_2_O (Fig. 3b). The ratio of average photon yields in CB[7] over H_2_O of 1.26 is somewhat lower than the value observed in bulk measurements (1.42), and is possibly explained by a more limited accessibility of the immobilised dye to CB[7] at the glass surface.

**Fig. 3:**
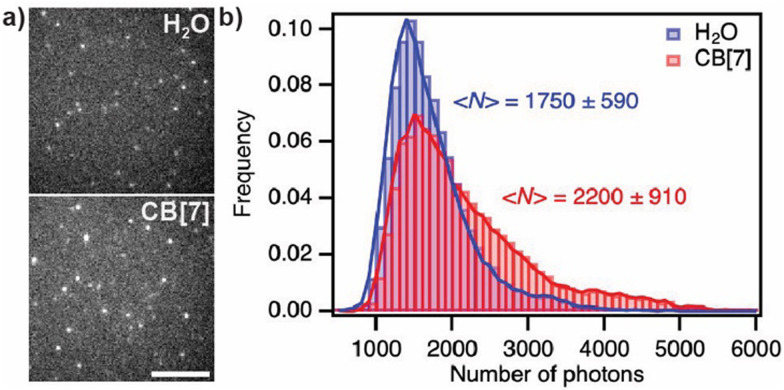
(a) Representative frames (identical contrast settings) of movies of single fluorescent ATTO655 molecules immobilised on a glass surface recorded in H_2_O or in H_2_O with 1 mM CB[7]. Scale bar: 5 μm. (b) Distribution of photons detected per localisation and per frame (sample size: 16 284 localisations in H_2_O, 17 347 with CB[7]).

Encouraged by these results, we tested whether encapsulation by CB[7] also led to an increased number of detected photons per localisation and per frame in SMLM under dSTORM conditions. We stained microtubules of fixed HeLa cells by immunofluorescence and imaged them in H_2_O containing 50 μM ascorbic acid^22^ and 1 mM of CB[7] (and otherwise no salts), with features below the diffraction limit being clearly resolved (Fig. 4a). A comparison with samples imaged under the same conditions but without CB[7] showed that the samples with CB[7] appeared brighter. The distribution of photons detected per localisation and per frame (Fig. 4b) was broader in the presence of CB[7] than in water and the average number of photons higher (3080 ± 1760 with CB[7] *vs.* 2330 ± 1130 in H_2_O). The localisation precision in samples imaged with CB[7] improved accordingly (Fig. 4c).

**Fig. 4:**
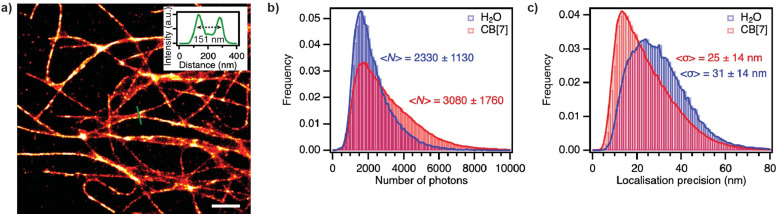
(a) Single-molecule localisation microscopy image of tubulin in fixed immunostained HeLa cells under dSTORM conditions and in the presence of a 1 mM concentration of CB[7]. Scale bar: 1 μm. The inset shows the cross-section indicated by a green line, resolving features below the diffraction limit and with full width at half-maximum of the two crossing microtubules of 57 and 66 nm respectively. (b) Distribution of the number of photons detected per localisation and per frame and (c) distribution of the localisation precision from dSTORM experiments in fixed HeLa cells in H_2_O and with CB[7] at a 1 mM concentration. The localisation precision was estimated using the procedure by Mortensen *et al.*^32^

In conclusion, we demonstrate that the red-emitting oxazines ATTO655, ATTO680, and ATTO700 bind to CB[7] with high affinity, thereby becoming more fluorescent, and that super-resolution experiments with ATTO655 benefit from the addition of CB[7] to the imaging medium. Encapsulation by CB[7] indeed leads to brighter fluorophores and an improved localisation precision owing to prevention of fluorescence quenching by water. Using host–guest interactions with CB[7] seems like a viable strategy to fine-tune and improve super-resolution imaging experiments.^33,34^

LB, JM and AF designed research. LB and AF performed research and analysed data. JM performed initial experiments and trained LB. AF wrote the paper. All authors commented on the final version of the manuscript.

We thank Claude Piguet and Amina Benchohra for advice and support with synthetic and characterisation procedures, as well as Ulf Rosspeintner for help with the TCSPC setup. This work was financially supported by the University of Geneva, the Swiss National Science Foundation (project no. 205321_207482), and the Société académique de Genève.

## Data availability

The data supporting this article have been included as part of the ESI.†

## Conflicts of interest

There are no conflicts to declare.

## Supplementary Material

CC-060-D4CC05274A-s001

## References

[cit1] Lavis L. D. (2017). Biochemistry.

[cit2] Grimm J. B., Tkachuk A. N., Xie L., Choi H., Mohar B., Falco N., Schaefer K., Patel R., Zheng Q., Liu Z., Lippincott-Schwartz J., Brown T. A., Lavis L. D. (2020). Nat. Methods.

[cit3] Fürstenberg A., Heilemann M. (2013). Phys. Chem. Chem. Phys..

[cit4] Heilemann M., van de Linde S., Schuttpelz M., Kasper R., Seefeldt B., Mukherjee A., Tinnefeld P., Sauer M. (2008). Angew. Chem., Int. Ed..

[cit5] Rust M. J., Bates M., Zhuang X. (2006). Nat. Methods.

[cit6] Betzig E., Patterson G. H., Sougrat R., Lindwasser O. W., Olenych S., Bonifacino J. S., Davidson M. W., Lippincott-Schwartz J., Hess H. F. (2006). Science.

[cit7] Hess S. T., Girirajan T. P., Mason M. D. (2006). Biophys. J..

[cit8] Sharonov A., Hochstrasser R. M. (2006). Proc. Natl. Acad. Sci. U. S. A..

[cit9] Jungmann R., Avendaño M. S., Woehrstein J. B., Dai M., Shih W. M., Yin P. (2014). Nat. Methods.

[cit10] Thompson R. E., Larson D. R., Webb W. W. (2002). Biophys. J..

[cit11] Lee S. F., Vérolet Q., Fürstenberg A. (2013). Angew. Chem., Int. Ed..

[cit12] Klehs K., Spahn C., Endesfelder U., Lee S. F., Fürstenberg A., Heilemann M. (2014). ChemPhysChem.

[cit13] Maillard J., Klehs K., Rumble C., Vauthey E., Heilemann M., Fürstenberg A. (2021). Chem. Sci..

[cit14] Dsouza R. N., Pischel U., Nau W. M. (2011). Chem. Rev..

[cit15] Jiang T., Qu G., Wang J., Ma X., Tian H. (2020). Chem. Sci..

[cit16] Maillard J., Rumble C. A., Fürstenberg A. (2021). J. Phys. Chem. B.

[cit17] Mohanty J., Nau W. M. (2005). Angew. Chem., Int. Ed..

[cit18] Nau W. M. , Hennig A. and Koner A. L., Squeezing Fluorescent Dyes into Nanoscale Containers—The Supramolecular Approach to Radiative Decay Engineering, Springer Series on Fluorescence, 2008, vol. 4, pp. 185–211

[cit19] Nau W. M., Mohanty J. (2005). Int. J. Photoenergy.

[cit20] Koner A. L., Nau W. M. (2007). Supramol. Chem..

[cit21] Heilemann M., van de Linde S., Mukherjee A., Sauer M. (2009). Angew. Chem., Int. Ed..

[cit22] Vogelsang J., Cordes T., Forthmann C., Steinhauer C., Tinnefeld P. (2009). Proc. Natl. Acad. Sci. U. S. A..

[cit23] Jana S., Nevskyi O., Höche H., Trottenberg L., Siemes E., Enderlein J., Fürstenberg A., Wöll D. (2024). Angew. Chem., Int. Ed..

[cit24] Trumpp M., Oliveras A., Gonschior H., Ast J., Hodson D. J., Knaus P., Lehmann M., Birol M., Broichhagen J. (2022). Chem. Commun..

[cit25] Straus O. H., Goldstein A. (1943). J. Gen. Physiol..

[cit26] Jarmoskaite I., AlSadhan I., Vaidyanathan P. P., Herschlag D. (2020). eLife.

[cit27] Shoichet B. K. (2006). J. Med. Chem..

[cit28] Mohanty J., Bhasikuttan A. C., Nau W. M., Pal H. (2006). J. Phys. Chem. B.

[cit29] Zhang S., Grimm L., Miskolczy Z., Biczok L., Biedermann F., Nau W. M. (2019). Chem. Commun..

[cit30] Marquez C., Nau W. M. (2001). Angew. Chem., Int. Ed..

[cit31] Mohanty J., Nau W. M. (2004). Photochem. Photobiol. Sci..

[cit32] Mortensen K. I., Churchman L. S., Spudich J. A., Flyvbjerg H. (2010). Nat. Methods.

[cit33] Sasmal R., Das Saha N., Schueder F., Joshi D., Sheeba V., Jungmann R., Agasti S. S. (2019). Chem. Commun..

[cit34] Kim D., Bossi M. L., Belov V. N., Hell S. W. (2024). Angew. Chem., Int. Ed..

