# Supplementary material for: Super-resolution imaging with a cucurbituril-encapsulated fluorophore
Source: Chem Commun (Camb). 2024 Nov 4;60(94):13943–6. doi: 10.1039/d4cc05274a (PMC11563205; doi:10.1039/d4cc05274a)
Supplement: CC-060-D4CC05274A-s001 [file CC-060-D4CC05274A-s001.pdf]

Supplementary Information (SI) for ChemComm.
This journal is © The Royal Society of Chemistry 2024

Supplementary Information

Super-Resolution Imaging with a Cucurbituril-Encapsulated Fluorophore

Liza Briant,[a,b] Jimmy Maillard,[a,b] and Alexandre Fürstenberg*,[a,b]

[a]Department of Physical Chemistry and [b]Department of Inorganic and Analytical Chemistry, University of Geneva, 1211 Geneva, Switzerland

alexandre.fuerstenberg@unige.ch

Contents

1. Experimental Section page S2

2. Supplementary Figures S6

3. Supplementary Table S12

4. Supplementary References S13

1. Experimental Section

Materials. ATTO655-NHS, ATTO655-COOH, ATTO680-COOH, and ATTO700-COOH, were purchased from ATTO-Tec. Stock solutions of the dyes were prepared at 1-10 mM concentration in DMSO and kept in the dark at –20 °C. H_2O was purified using a Milli-Q system (Millipore). Phosphate buffer saline (PBS, pH 7.4; composition: NaCl 155.17 mM, KH_2PO_4 1.06 mM, Na_2HPO_4 2.97 mM) was from Life Technologies.

CB[7] was synthesised following the procedure of Nau and coworkers.[S1] Briefly, 11.4 g of glycoluril (80.2 mmol) were mixed with 60 mL of cold 9 M sulphuric acid (540 mmol). 14 mL of 37% formaldehyde (186 mmol) were slowly added and the mixture was stirred until a gel formed. The temperature was then increased to 100 °C and the reaction was let to run for 72 h under reflux. The reaction mixture was then poured into 200 mL of milliQ water and 800 mL of acetone, which led to the precipitation of all cucurbit[*n*]urils and oligomers. The precipitate was collected and washed with 1.5 L of an acetone/milliQ water (8:2 v/v) mixture. The solid was added to 400 mL of milliQ water to dissolve all cucurbit[*n*]urils except CB[6]. The latter was collected by filtration and 300 mL of acetone were added to the resulting filtrate to induce the precipitation of the main fraction of CB[7]. The precipitate was removed, dissolved in 220 mL of a 10:1 milliQ water/acetone mixture to precipitate any remaining CB[6] and other glycoluril oligomers. This solid was discarded. The major fraction of CB[7] was precipitated by further adding 100 mL of acetone. CB[7] was then filtered and an amorphous yellow solid was obtained, dissolved again in milliQ water and lyophilised, leading to a white fluffy solid. ^{1}H NMR (400 MHz, D_2O) δ 4.26 − 4.22 (d, ^2J = 15.39, 1H), δ 5.54 (s, 1H), δ 8.82 – 5.78 (d, ^2J = 15.44, 1H). ^{13}C NMR (101 MHz, D_2O) δ 52.47, δ 71.18, δ 156.40. ESI-MS (positive mode – H_2O + TFA): *m*/*z*: 1163.3 $[M+H]^+$, 582.2 $[M+2H^+]^{2+}$. NMR assignments and spectra are provided in Figures S6-S10.

Steady-state photophysics. Absorption spectra were recorded on a Cary 50 spectrophotometer. Fluorescence spectra were recorded on a FluoroMax-4 (Horiba Jobin Yvon) fluorescence spectrometer (R2658 detector from Hammamatsu) and corrected for the wavelength dependence of the detector. Spectra were taken using disposable acrylic 1-cm cuvettes (Sarstedt). Fluorescence quantum yields were determined from the integrated fluorescence spectra relatively to the corresponding free dyes in water whose quantum yields are known.[S2] The uncertainty on absolute quantum yield values is estimated to ±10%.

Fluorescence lifetime measurements. Fluorescence lifetime measurements were performed under magic angle conditions using the time-correlated single-photon counting (TCSPC) technique. Excitation was provided by a tuneable-pulsed picosecond source (NKT Photonics, SuperK EXTREME). The excitation light was focused onto the sample (1-cm disposable acrylic cuvette, Sarstedt) using a Schwarzschild objective. The fluorescence emission from the sample was collected into an optical fibre, spectrally filtered using a spectrograph (Triax 190, Horiba) and detected on a photomultiplier tube (PMA 192C, PicoQuant). All excited-state lifetimes were extracted from the measured traces by iterative reconvolution of a trial function (a single or double exponential function) with the measured instrument response function. The wavelength-dependent instrument response function had a full width at half-maximum of ~130 ps. The uncertainty on excited-state lifetimes is estimated to ± 0.05 ns.

For time-resolved fluorescence polarisation anisotropy measurements, two fluorescence traces with the polarisation analyser set either parallel or perpendicular to the excitation beam polarisation were recorded. Anisotropy decays were then reconstructed by using standard procedures, with G-factors being determined by tail matching. The decay of the fluorescence polarisation anisotropy with time, $r(t)$, was modeled with a single exponential function of the type $r(t) = r_0 \cdot \exp(-t/\tau_r)$, where r_0 is the initial anisotropy and τ_r the rotational correlation time.

Sample preparation and binding equilibria. Host-guest complexes between the dyes and CB[7] were prepared by mixing adequate volumes of CB[7] stock solutions freshly prepared in pure H_2O before use with dye stock solutions. The final dye concentration was kept constant within an experimental series between 1 and 2 μM. The binding equilibrium of fluorophores to CB[7] was analysed using a 1:1 binding isotherm for the general reaction $F + CB \rightleftharpoons FCB$, where F designates a fluorophore molecule, CB the CB[7] cage, and FCB the 1:1 complex between F and CB. The relationship between the fraction of bound fluorophore x_{FCB} (measured through a fluorescence observable such as the quantum yield or lifetime), the dissociation constant K_d, and the total fluorophore and CB[7] concentrations, c(F) and c(CB) respectively, is given by:[S3]

$$x_{FCB} = \frac{K_d + c(F) + c(CB) - \sqrt{(K_d + c(F) + c(CB))^2 - 4 \cdot c(F) \cdot c(CB)}}{2 \cdot c(F)} \qquad (S1)$$

Water accessibility. As detailed elsewhere, the excited-state lifetime of the dyes ATTO655, ATTO680, and ATTO700 can be directly related to the number of water molecules in their contact sphere as H_2O causes fluorescence quenching of these dyes.[S2, S4] The residual water

quenching efficiency, f_q, which is also directly the water accessibility to the dye, can be estimated from the following relationship:[S4]

$$f_q = \frac{N_{H_2O,i}}{N_{max}} = \frac{\tau_{fl,i}^{-1} - \tau_{D_2O}^{-1}}{\tau_{H_2O}^{-1} - \tau_{D_2O}^{-1}} \qquad (S2)$$

where $N_{H2O,i}$ is the number of remaining H_2O molecules in the first solvent sphere of the dye in the environment of interest i, N_{max} the number of H_2O molecules in the first solvent sphere in pure H_2O, $\tau_{fl,i}$ the excited-state lifetime of the fluorophore in the environment i, τ_{D2O} the excited-state lifetime of the fluorophore in pure D_2O (taken as an entirely non-quenching environment), and τ_{H2O} the excited-state lifetime of the fluorophore in pure H_2O. The values of the lifetimes in pure H_2O and pure D_2O were taken from Maillard et al.[S2]

Single-molecule immobilisation. Single ATTO655-NHS molecules were immobilised on the glass surface of a glass-bottom 96-well plate (Greiner #655892) that we modified with (3-glycidyloxypropyl)trimethoxysilane (GOPS, Sigma-Aldrich). A fresh GOPS solution was prepared by adding 10 µL of GOPS to 460 µL of pure ethanol, 25 µL of pure water, and 5 µL of acetic acid. 50 µL of this solution were deposited into the desired wells and left to react for 60 minutes at room temperature. The wells were then washed three times with a 7:3 (v/v) ethanol-water mixture. ATTO655-NHS was coupled to the surface by depositing 100 µL of 1-5 nM solutions in water into each well and leaving the coupling reaction to proceed for 90 minutes at room temperature. The wells were subsequently washed three times with MilliQ water. Imaging in the wells was performed with 200 µL of either H_2O or of a 1 mM CB[7] solution in H_2O.

Cell culture. HeLa cells were cultured at 37 °C with 5% CO_2 in phenol red free RPMI supplemented with 10% foetal bovine serum, 100 units/ml penicillin, and 100 µg/ml streptomycin. All cell culture reagents were purchased from ThermoFisher Scientific.

Immunofluorescence of microtubules. For single-molecule imaging experiments, HeLa cells were seeded on an 8-well chambered coverglass (Nunc Lab-Tek, ThermoFisher Scientific) coated with 1 µg/ml of fibronectin (PanReac Applichem) in full medium for 45 min at 37 °C and rinsed with PBS. They were left to grow for 48 hours. Cells were then treated with microtubule stabilisation buffer (80 mM PIPES pH 6.8, 1 mM $MgCl_2$; 5 mM EGTA, 0.5% (v/v) Triton X-100) for 30 seconds, washed with PBS, and fixed with 4% (v/v) paraformaldehyde in PBS for 20 min at 4 °C. Free aldehydes were quenched for 15 minutes by treatment with

50 mM NH_4Cl. The samples were then washed three times for 5 minutes with PBS. The samples were subsequently incubated first with immunofluorescence (IF) staining buffer (PBS pH 7.4 with 1% (w/v) BSA and 0.1% (v/v) Triton X-100) for 10 minutes and then with a 1 μg/mL mouse anti-β-tubulin primary monoclonal antibody 2-28-33 (#32-2600, Invitrogen) solution in the same buffer for 1 hour. After that, the cells were washed four times with PBS containing 0.1% (v/v) Triton X-100 (PBST). Secondary antibody staining was performed with 1 μg/mL of ATTO 655-labeled P1-goat anti-mouse antibody (#50283, Sigma-Aldrich) in IF buffer for 1 hour. Cells were washed four times with PBST and four times with PBS. The samples were stored dry at 4 °C in the dark.

Single-molecule and dSTORM imaging. For single-molecule and dSTORM imaging, immobilised ATTO655 molecules and fixed cell samples were put into either pure H_2O or a 1 mM CB[7] solution in H_2O supplemented with freshly prepared 50 μM ascorbic acid[S5]. Single-molecule and dSTORM movies were acquired on an Olympus IX71 inverted epi-fluorescence microscope equipped with an oil-immersion objective (Olympus, Universal Plan Apochromat, 60 x, 1.50 NA), a motorised stage (Applied Scientific Instruments, MS-2000), a piezo z-stage (Piezoconcept, Z-INSERT500), and a focus stabilisation system (Applied Scientific Instruments, Crisp). An Abbelight SAFe180 module was attached to the side C-mount of the microscope body. It received fibre-coupled laser excitation from a 500 mW Oxxius LaserBoxx DPSS 640 nm laser (LPX-640L-500) through an ASTER (Adaptable Scanning for Tunable Excitation Regions) scanning module[S6] (Abbelight). Excitation and fluorescence light were separated using a quinta-band dichroic mirror (Chroma 89903bs) and emission light was further filtered by a quinta-band bandpass filter (Chroma ZET405/445/514/561/640). The fluorescence was detected on a Teledyne Photometrix Kinetix sCMOS camera with 15 ms exposure time for dSTORM imaging and 50 ms for regular single-molecule imaging. Each camera pixel corresponded to a 108x108 nm^2 square in the sample plane.

Single-molecule and dSTORM data processing. Single-molecule and dSTORM movies were analysed with the Picasso software (v0.7.0).[S7] Molecules were localised with Picasso Localise using the following parameters: baseline 99.5 photons, sensitivity 0.501, quantum efficiency 0.90, pixel size 108 nm, and a gradient of 5000 with an MLE algorithm. Super-resolved images were reconstructed with Picasso Render. The image stacks were drift corrected using redundant cross correlation (RCC) on Picasso Render with 500-1000 frames. The number of photons were extracted directly from Picasso Localise. The localisation precision was estimated using the procedure by Mortensen et al.[S8] using a point-spread function of 1 pixel.

2. Supplementary Figures

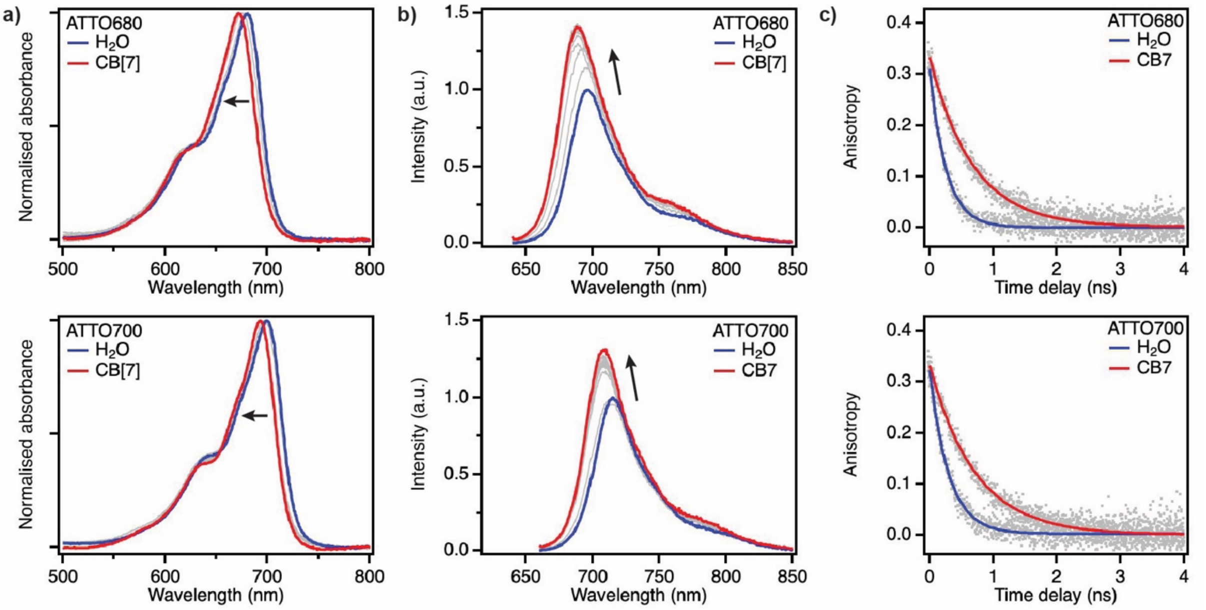

Figure S1. (a) Intensity-normalised absorption spectra and (b) fluorescence spectra of ATTO680, and ATTO700 in H_2O (blue traces) and in the presence of various (1.4–667 μM) concentrations of CB[7] (grey traces) up to 1 mM (red traces). (c) Decay of the fluorescence polarisation anisotropy of the same dyes in pure H_2O and in the presence of 1 mM of CB[7]. Solid lines represent best monoexponential fits to the data points (grey).

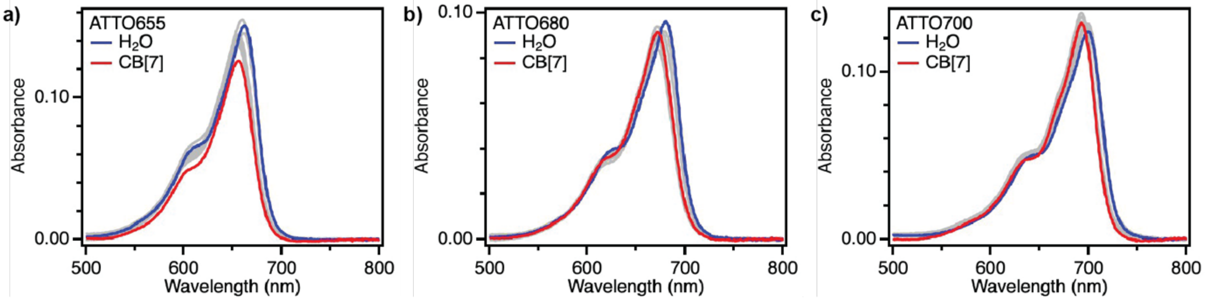

Figure S2. Absorption spectra of ATTO655, ATTO680, and ATTO700 in H_2O (blue traces) and in the presence of various (1.4–667 μM) concentrations of CB[7] (grey traces) up to 1 mM (red traces).

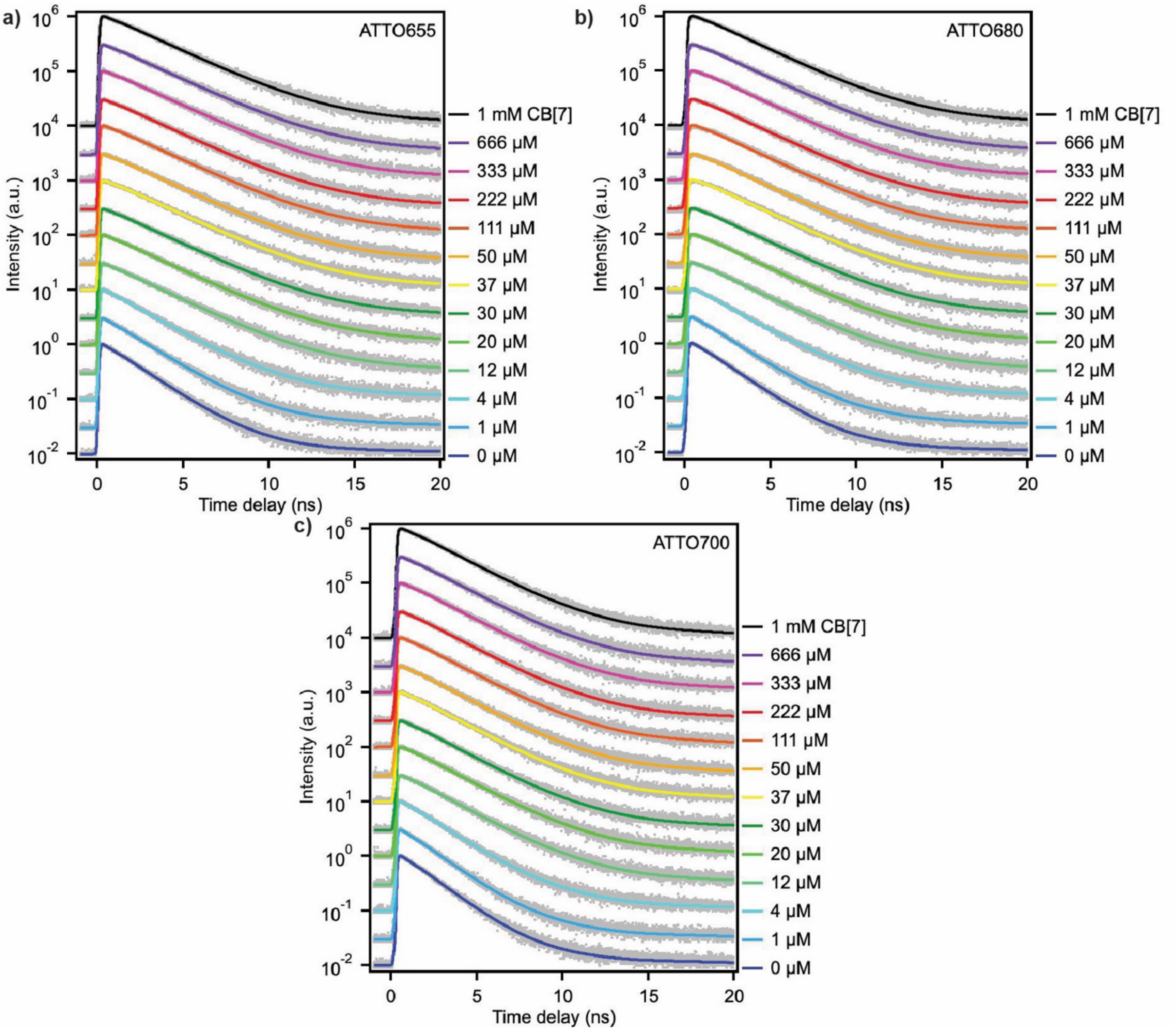

Figure S3. Intensity-normalised fluorescence time profiles of the investigated fluorophores in the presence of various concentrations of CB[7] and in pure H_2O (bottom trace for every dye). Solid black lines are exponential fits to the data points. The traces are shifted on the vertical axis for clarity.

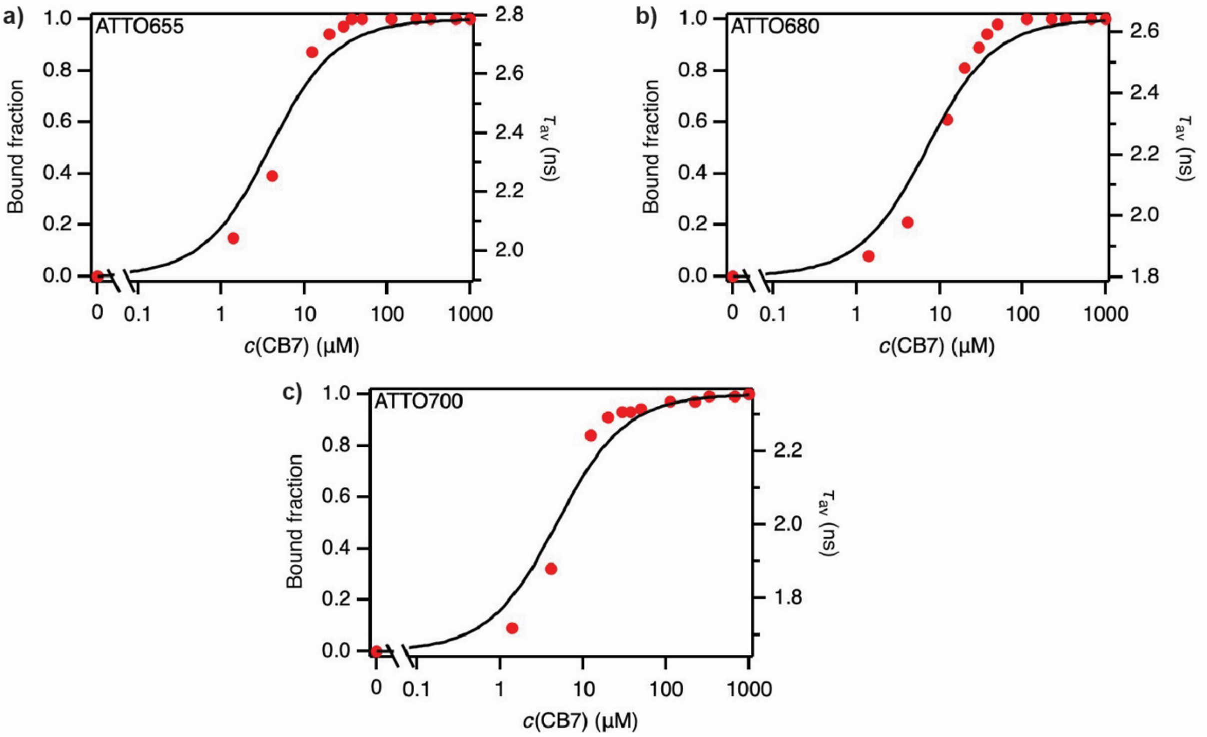

Figure S4. Bound fraction (left axis) and amplitude-weighted average fluorescence lifetime (τ_{av}, right axis) of the investigated dyes as a function of the total CB[7] concentration in H_2O. Solid lines represent fits to the data points with a 1:1 binding isotherm. In all cases, the bound fraction is obtained by calculating the ratio $a_2/(a_1+a_2)$ using the amplitudes of the fluorescence decay kinetics in Table S1.

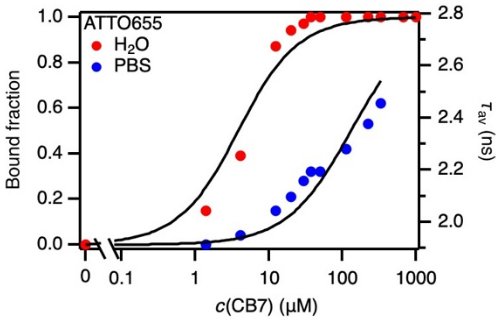

Figure S5. Bound fraction and amplitude-weighted average fluorescence lifetimes (τ_{av}, right axis) of the investigated dyes as a function of the total CB[7] concentration in H_2O or in PBS. CB[7] started to precipitate at concentrations above 333 μM in PBS. Solid lines represent fits to the data points with a 1:1 binding isotherm, yielding an estimated K_d value in PBS of $(130 \pm 20)\cdot10^{-6}$.

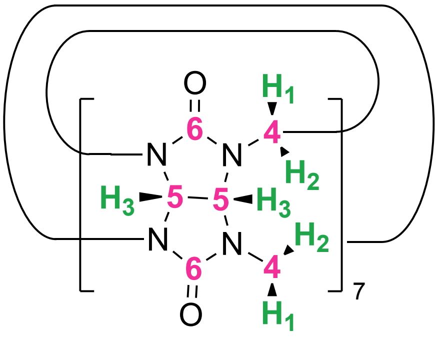

Figure S6. Assignment of the H and C atoms in NMR spectra of CB[7].

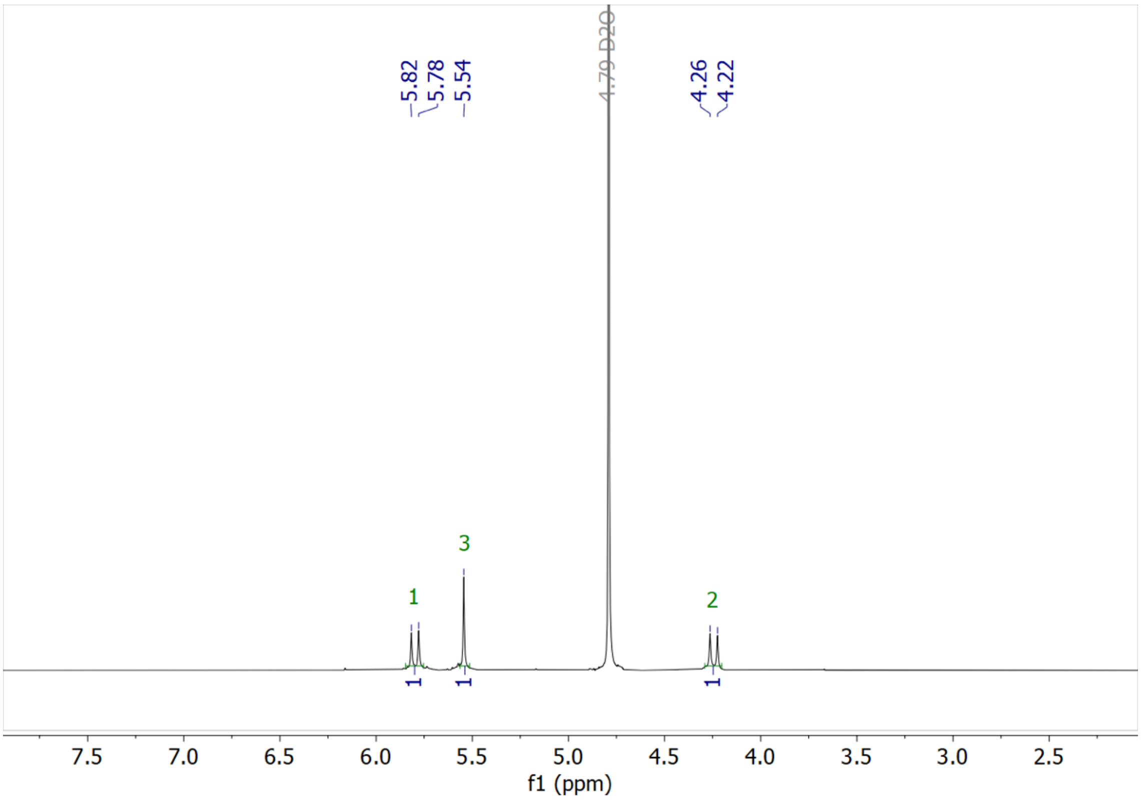

Figure S7. ^{1}H NMR spectrum of CB[7] in D_2O (298 K).

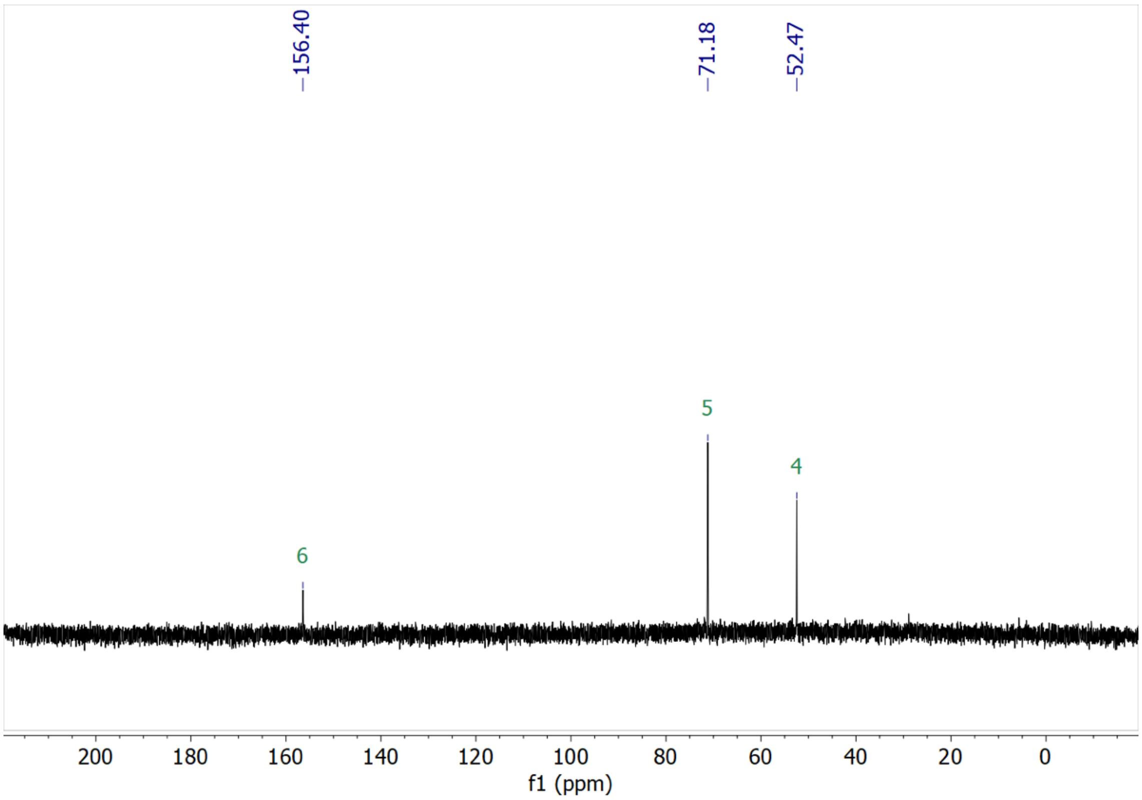

Figure S8. ^{13}C NMR spectrum of CB[7] in D_2O (298 K).

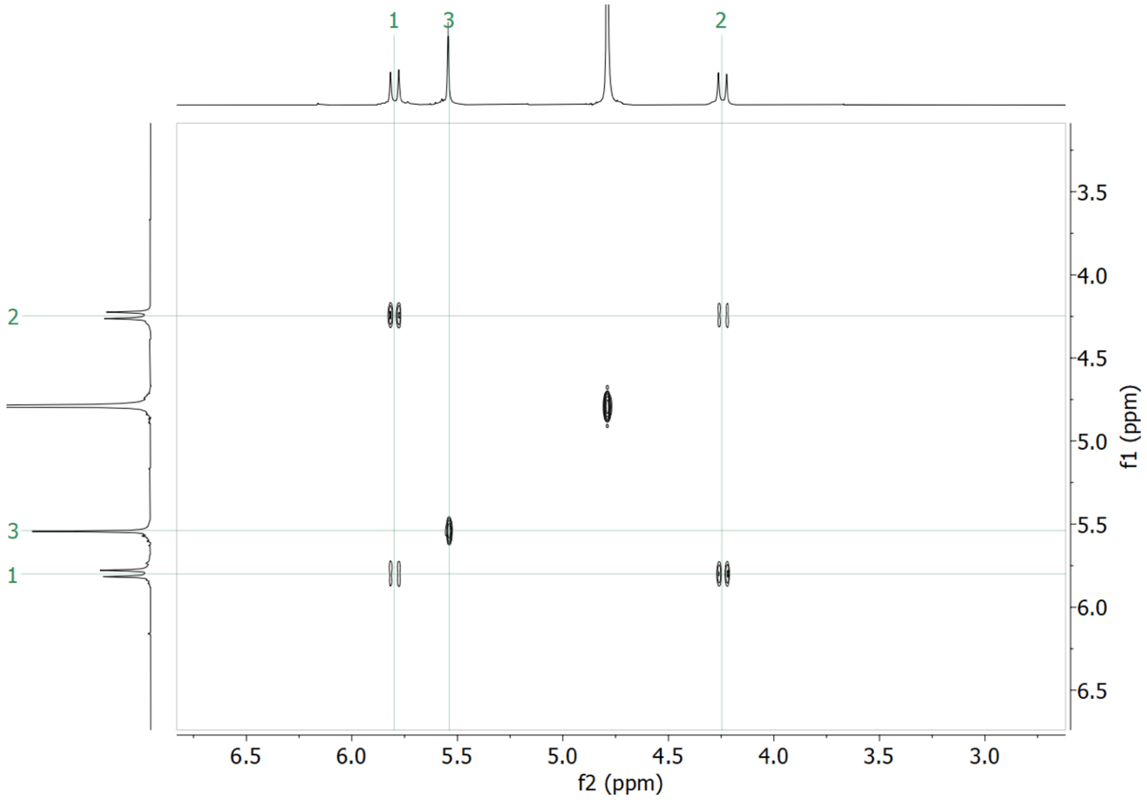

Figure S9. ^{1}H –^{1}H COSY NMR spectrum of CB[7] in D_2O (298 K).

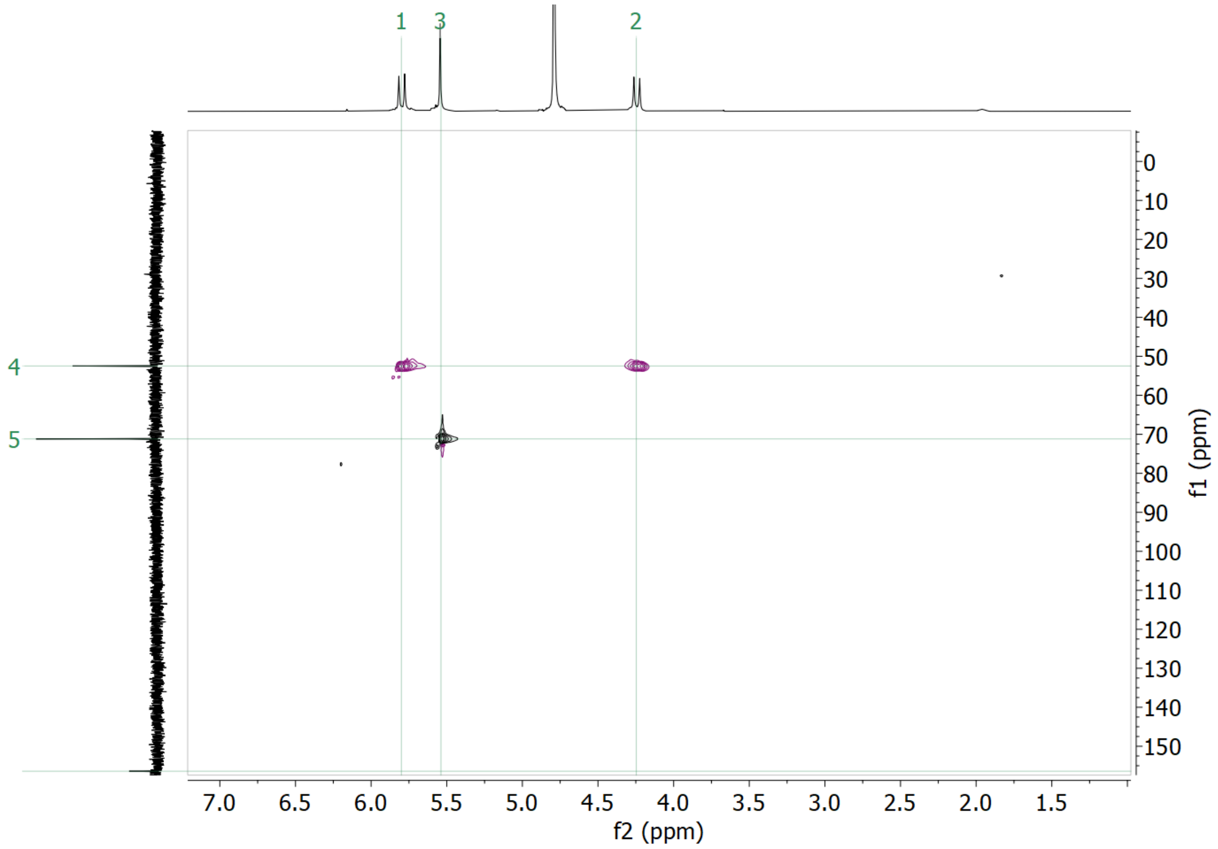

Figure S10. $^{1}H-^{13}C$ HSQC NMR spectrum of CB[7] in D_2O (298 K).

3. Supplementary Table

Table S1. Absolute (Φ_{fl}) and relative ($\Phi_{fl,rel}$) fluorescence quantum yield of the dyes ATTO655, ATTO680, and ATTO700 in pure H_2O and in the presence of various concentrations of CB[7], and results of a global analysis of the fluorescence decays of the dyes (Figure S3) in the presence of various concentrations of CB[7] (c(CB[7])) with a biexponential function, where τ_1 and τ_2 are the lifetimes of the two components representing the free dye in H_2O and the dye bound to CB[7], respectively, and a_1 and a_2 their relative amplitudes. Uncertainties on the fluorescence quantum yields are estimated to ±10%, uncertainties on the lifetimes to ±0.05 ns.

	**ATTO655**				**ATTO680**				**ATTO700**			
	**$\Phi_{fl}(H_2O)^a$**	**$\Phi_{fl}(CB[7])^b$**	**τ_1 (ns)**	**τ_2 (ns)**	**$\Phi_{fl}(H_2O)^a$**	**$\Phi_{fl}(CB[7])^b$**	**τ_1 (ns)**	**τ_2 (ns)**	**$\Phi_{fl}(H_2O)^a$**	**$\Phi_{fl}(CB[7])^b$**	**τ_1 (ns)**	**τ_2 (ns)**
	0.28	0.40	1.91	2.79	0.30	0.44	1.80	2.68	0.25	0.34	1.64	2.36
**c(CB[7]) (mM)**	**$\Phi_{fl,rel}^c$**	**τ_{av} (ns)^d**	**a_1**	**a_2**	**$\Phi_{fl,rel}^c$**	**τ_{av} (ns)^d**	**a_1**	**a_2**	**$\Phi_{fl,rel}^c$**	**τ_{av} (ns)^d**	**a_1**	**a_2**
0	1.00	1.91	1.00	0.00	1.00	1.80	1.00	0.00	1.00	1.64	1.00	0.00
0.0014	1.00	2.04	0.85	0.15	1.17	1.91	0.92	0.13	0.98	1.71	0.91	0.09
0.0041	1.07	2.25	0.61	0.39	1.34	2.19	0.79	0.44	1.04	1.87	0.68	0.32
0.012	1.26	2.68	0.13	0.87	1.46	2.57	0.39	0.87	1.19	2.24	0.16	0.84
0.020	1.36	2.74	0.06	0.94	1.43	2.63	0.19	0.94	1.23	2.29	0.09	0.91
0.030	1.36	2.76	0.03	0.97	1.46	2.64	0.11	0.95	1.24	2.30	0.07	0.93
0.037	1.35	2.79	0.00	1.00	1.46	2.68	0.00	1.00	1.23	2.31	0.07	0.93
0.050	1.35	2.79	0.00	1.00	1.46	2.68	0.00	1.00	1.27	2.32	0.06	0.94
0.11	1.36	2.79	0.00	1.00	1.44	2.68	0.00	1.00	1.24	2.33	0.03	0.97
0.22	1.35	2.79	0.00	1.00	1.48	2.68	0.00	1.00	1.28	2.33	0.03	0.97
0.33	1.33	2.79	0.00	1.00	1.34	2.68	0.00	1.00	1.25	2.35	0.01	0.99
0.67	1.45	2.79	0.00	1.00	1.40	2.68	0.00	1.00	1.23	2.35	0.01	0.99
1.0	1.42	2.79	0.00	1.00	1.47	2.68	0.00	1.00	1.34	2.36	0.00	1.00

[a]From ref. S2. [b]Measured in H_2O in the presence of 1 mM CB[7]. [c]The free dye in H_2O is used as a reference. [d]Amplitude-weighted average fluorescence lifetime.

4. Supplementary References

S1 C. Marquez, F. Huang and W. M. Nau, *IEEE Trans. Nanobiosci.*, 2004, **3**, 39-45.

S2 J. Maillard, K. Klehs, C. Rumble, E. Vauthey, M. Heilemann and A. Fürstenberg, *Chem. Sci.*, 2021, **12**, 1352-1362.

S3 I. Jarmoskaite, I. AlSadhan, P. P. Vaidyanathan and D. Herschlag, *eLife*, 2020, **9**, e57264.

S4 J. Maillard, C. A. Rumble and A. Fürstenberg, *J. Phys. Chem. B*, 2021, **125**, 9727-9737.

S5 J. Vogelsang, T. Cordes, C. Forthmann, C. Steinhauer and P. Tinnefeld, *Proc. Natl. Acad. Sci. USA*, 2009, **106**, 8107-8112.

S6 A. Mau, K. Friedl, C. Leterrier, N. Bourg and S. Lévêque-Fort, *Nat. Commun.*, 2021, **12**, 3077.

S7 J. Schnitzbauer, M. T. Strauss, T. Schlichthaerle, F. Schueder and R. Jungmann, *Nat. Protoc.*, 2017, **12**, 1198-1228.

S8 K. I. Mortensen, L. S. Churchman, J. A. Spudich and H. Flyvbjerg, *Nat. Methods*, 2010, **7**, 377-381.
